# Leveraging the vantage point – exploring nurses’ perception of residents’ communication skills: a mixed-methods study

**DOI:** 10.1186/s12909-023-04114-6

**Published:** 2023-03-03

**Authors:** Komal Abdul Rahim, Maryam Pyar Ali Lakhdir, Noreen Afzal, Asma Altaf Hussain Merchant, Namra Qadeer Shaikh, Ali Aahil Noorali, Umar Tariq, Rida Ahmad, Saqib Kamran Bakhshi, Saad bin Zafar Mahmood, Muhammad Rizwan Khan, Muhammed Tariq, Adil H. Haider

**Affiliations:** 1 Dean’s Office, Medical College, Aga Khan University, Stadium Road, P. O. Box 3500, Karachi, 74800 Pakistan; 2 Department of Community Health Sciences, Aga Khan University, Stadium Road, P. O. Box 3500, Karachi, 74800 Pakistan; 3 Internal Medicine, Aga Khan University, Stadium Road, P. O. Box 3500, Karachi, 74800 Pakistan; 4 Medical College, Aga Khan University, Karachi, Pakistan

**Keywords:** Communication, Nurses, Residents

## Abstract

**Introduction:**

Effective communication is key to a successful patient-doctor interaction and improved healthcare outcomes. However, communication skills training in residency is often subpar, leading to inadequate patient-physician communication. There is a dearth of studies exploring the observations of nurses – key members of healthcare teams with a special vantage point to observe the impact of residents’ communication with patients. Thus, we aimed to gauge the perceptions of nurses regarding residents’ communication skills expertise.

**Methods:**

This study employed a sequential mixed-methods design, and was conducted at an academic medical center in South Asia. Quantitative data was collected via a REDCap survey using a structured validated questionnaire. Ordinal logistic regression was applied. For qualitative data, In-depth interviews were conducted with nurses using a semi-structured interview guide.

**Results:**

A total of 193 survey responses were obtained from nurses hailing from various specialties including Family Medicine (*n* = 16), Surgery (*n* = 27), Internal Medicine (*n* = 22), Pediatrics (*n* = 27), and Obstetrics/Gynecology (*n* = 93). Nurses rated long working hours, infrastructural deficits, and human failings as the main barriers to effective patient-resident communication. Residents working in in-patient settings were more likely to have inadequate communication skills (*P*-value = 0.160). Qualitative data analysis of nine in-depth interviews revealed two major themes: existing status-quo of residents’ communication skills (including deficient verbal and non-verbal communication, bias in patient counselling and challenging patients) and recommendations for improving patient-resident communication.

**Conclusion:**

The findings from this study highlight significant gaps in patient-resident communication from the perception of nurses and identify the need for creating a holistic curriculum for residents to improve patient-physician interaction.

**Supplementary Information:**

The online version contains supplementary material available at 10.1186/s12909-023-04114-6.

## Key messages

**What is already known about the topic?**

Effective communication is the cornerstone of any interaction that healthcare professionals have with patients. Many studies have been conducted to assess the communication skills of healthcare professionals. However, none of the studies conducted in Pakistan have taken a holistic viewpoint of partner healthcare professionals such as nurses, to assess the communication skills of residents who primarily provide information to the patients. Furthermore, the current residency programs in Pakistan do not adequately consider communication skills curricula, mandating the need for educational modules to be an integral part of the post-graduate residency program.

**What this study adds?**

To the best of our knowledge, this is the first mixed-method study that undertook the viewpoint of nurses regarding residents' communication skills. Nurses leverage a special vantage point since they are the frontline care providers and in a better place to witness patient-physician interaction. The findings from this study provide insight of nurses regarding standing of communication skills and how they can be further improved.

**How this study might affect research, practice or policy?**

The nurses in our study highlighted that the current communication skills of residents are inadequate for effective patient-physician interaction. Nurses suggested that a communication skills curriculum introduced during the residency program might help in filling the gap. The center of focus may include breaking bad news, simplifying medical terminologies and eradicating the effect of implicit bias while interacting with patients.

## Introduction

Liaison between residents and other healthcare professionals, especially nurses, is an important predictor of improved patient outcomes [1]. Effective communication between patients and all members of the healthcare team including attendings, fellows, residents and nurses is essential for enhanced patient outcomes [2]. Nurses form an integral part of healthcare teams with a special vantage point for assessing patient-resident communication, since they work in close contact with both the residents and patients. Hence, they have a unique opportunity to directly observe residents providing patient care in critical aspects. Ogunyumi et al., in their study of over 1600 nurses evaluating patient-resident interactions, suggested that such evaluations could be important for giving formative feedback to residents for their professional development [3, 4].

Evidence suggests that patients judge their consultation experiences by how well the doctor interacted with them. Duration of the consultation, coupled with the tone and attitude of residents while interacting with patients are some of the aspects they greatly value to evaluate their overall experience [1, 5]. Improved health outcomes have been reported when a patient-doctor interaction goes beyond a monotonous history-taking session, with adequate time and effort invested into exploring the patient’s ideas and concerns [6, 7]. However, previous efforts exploring residents' communication skills expertise have consistently identified significant gaps including residents not introducing themselves to patients, frequently using medical jargons, [8] and perceiving communication with patients as a means of delivering information rather than a conversation [9].

Nurses’ communication skills are rated highest by other healthcare professionals as evidenced from the literature [10]. Hence, their perception regarding patient-resident communication can give direction on improvement strategies. In a study conducted to assess nurses’ perception of outstanding residents, nurses placed significant importance on residents’ communication skills, particularly those relating to patient counselling [11]. Moreover, evaluation of residents’ communication expertise by non-physicians such as nurses can be insightful, since they observe them in critical aspects of patient care that attending physicians do not routinely observe. This can result in a considerably different evaluation from the latter [12]. A study conducted in a tertiary care hospital in Pakistan employed multiple observers including nurses, faculty, and residents to rate patient-resident communication through surveys. Results showed that nurses rated residents lowest on communication skills as compared to other raters [13].

Given the many benefits of effective patient-doctor communication, and dearth of literature on nurses’ perspective on the topic, there is a dire need to understand how residents communicate with patients in these settings. Hence, this study aimed to explore the perception of nurses working in a large tertiary care hospital about residents’ communication skills with patients and recommendations for improvement.

## Methodology

### Study design, setting and duration

To delineate nurses’ perception regarding residents’ communication skills, we employed a sequential mixed-methods study design. A detailed methodology has been published previously [14]. This study was conducted at the largest academic medical center (AMC) in Pakistan which is accredited by the Accreditation Council for Graduate Medical Education (ACGME-I) for its residency programs. Quantitative data collection using a structured survey questionnaire was followed by in-depth interviews to explore the perceptions and observations of nurses regarding patient-resident communication. Total duration of the study was 3 months (July 2021 – September 2021).

### Study participants and sampling

Purposive sampling was employed to recruit nurses for both the quantitative and qualitative phases of the study. Nurses working within the departments of family medicine, surgery, medicine, pediatrics, and obstetrics and gynecology were eligible to take part. All nurses working in these departments for more than 6 months and gave consent to participate were included.

### Study instrument and data collection

#### Quantitative data

The questionnaire was adapted from a validated survey developed for self-assessment of residents’ communication skills [15]. (Supplemental file 1). It consists of components ranging from socio-demographics (including gender, age, primary institution of higher education, assigned specialty, frequency of interactions the nurses have with residents, and years of experience) to aspects of communication expertise. The items assessed in the latter domain include: A) patient-resident interactions and conflicts, B) verbal and non-verbal communication (including content, setting of discussion/interview sessions, breaking bad news, team dynamics) and C) barriers to practicing good communication skills.

Responses to all sections are based on a standard five-point Likert scale. For Section B, a higher score denotes better communication of the resident while a higher score in Section C suggests a significant barrier to practicing effective communication. The survey was designed on REDCap software and deployed electronically to nurses via their individual institutional email addresses. Follow-up email reminders were also sent. After obtaining all the responses for Section A through C, a composite communication score was calculated which indicated nurses’ views about residents’ communication skills.

#### Qualitative data

In-depth interviews (IDIs) were conducted with nurses using a semi-structured interview guide (Supplemental file 1). It was ensured that there was representation from all departments mentioned in the inclusion criteria. Extensive literature search and feedback from experts was used to develop this interview guide. Interview questions were kept open-ended to encourage a free flow of information. These questions were followed by probes to elicit information from the participants in detail. Questions centered around nurses’ perception of ideal patient-physician communication, their observations of residents’ verbal and non-verbal communication skills, barriers to practicing effective communication, and suggestions to improve the interaction between residents and patients. Institutional email addresses of the nurses were used to disseminate a sign-up form for participating in the interviews. Interviews were conducted by a research team member (NA) in Urdu over Zoom which lasted for 30 to 45 minutes. These were audio-recorded with participants’ consent. Data collection continued until thematic saturation was achieved [16].

#### Rigor

Multiple strategies were used to ensure methodological rigor. Prior to beginning the interviews, rapport was built with the respondents and confidentiality of data reassured. The interviewer made reflexive notes after each IDI to document the influence of their background and pre-conceived notions on the analytical process [17]. For establishing credibility of the findings, two types of triangulations were used. The first was investigator triangulation, whereby two researchers analyzed the qualitative data and the second being methodological triangulation which was achieved using a mixed-methods approach [18].

To integrate qualitative and quantitative data, we used a Joint Display (Refer to Table 4). This display links the quantitative results with qualitative themes, illustrating agreement and disagreement between the two perspectives.

### Data analysis

For quantitative data, STATA (version 16) was used to conduct the statistical analyses. For categorical variables, frequencies and percentages are reported such as those for demographic variables and barriers to residents’ practicing good communication. For continuous variables, means and standard deviations are reported with a *p*-value of < 0.05 considered significant. Since each of the 29 items in Section B were scored using a 5-point Likert scale, the minimum cumulative communication score was 29 and the maximum score was 145. Based on the mean and standard deviation, the combined scores were broken down into three categories. Scores less than 97 depicted inadequate communication skills, 97–140 depicted fair communication skills and > 140 score depicted adequate communication skills. These scores were then compared against each of the socio-demographic variables of nurses by estimating the crude odds ratio (OR) with 95% confidence interval using ordinal logistic regression. Socio-demographic variables that had a *p*-value of < 0.25 were entered in the stepwise adjusted logistic regression model.

For qualitative data, audio recordings of the interviews were transcribed verbatim and then translated into English. All participant identifiers and personal information was removed from the transcripts. These transcripts were then imported into NVivo (version 11, QSR International). Braun and Clark’s six-step method guided thematic analysis of the data [19]. Two members of the research team (NA, AAHM) initially read the transcripts multiple times to acquaint themselves with the data, followed by independent coding of the transcripts. Thereafter, the researchers met to discuss their individual codes and generate a final codebook based on a consensus. Similar codes were organized to generate themes and subthemes, followed by their detailed descriptions.

### Ethical considerations

This study obtained ethical approval from the Aga Khan University Ethical Review Committee (2021–6041-17126). As part of electronic data collection, an online version of the consent form was provided to the participants for both the survey and the interviews. Signing of the consent forms was mandatory for inclusion in the study. Data was secured on a password encrypted file and only those members of the team directly involved in analysis were granted access.

## Results

A total of 193 survey responses were obtained from the nursing staff of five specialties. Demographic characteristics of the participants are shown in Table 1. Majority of the respondents were females (86.39%), under 30 years of age (52.33%) and obtained their undergraduate training at private institutions (77.20%).

**Table 1:** Demographic characteristics of Quantitative Survey (*n*=192)

Characteristics	n (%)
**Gender**	
Female	165 (86.39)
Male	26 (13.61)
**Age**	
< 30 years	101 (52.33)
> 30 years	92 (47.67)
**Institution of Undergraduate Education**	
Private	149 (77.20)
Public	44 (22.8)
**Assigned Specialty**	
Family Medicine	16 (8.29)
Medicine	22 (11.4)
Obstetrics/Gynecology	93 (48.19)
Pediatrics	27 (13.99)
Surgery	27 (13.99)
Others	8 (4.15)
**Interaction of Frequency with residents/day (*N*** **= 193)**	
< 2 interactions	43 (22.28)
2–4 interactions	70 (36.27)
4–6 interactions	37 (19.17)
6–8 interactions	17 (8.81)
> 8 interactions	26 (13.47)
**Years of Experience (N = 193)**	
< 5 years	70 (36.27)
5–10 years	75 (38.86)
> 10 years	48 (24.87)

**Fig. 1:**
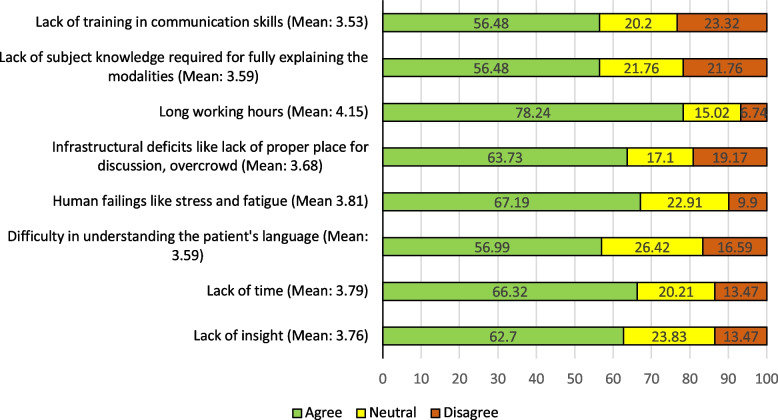
Barriers to residents practicing good communication skills

Figure 1 shows ratings of barriers to effective patient-resident communication by nurses. Long working hours was identified as the major hindrance to adequate patient-resident communication (78.24%). This was followed by infrastructural deficits such as lack of designated counseling spaces and overcrowding (63.73%), human failings (67.19%) and lack of time (66.32%).

Table 2 shows the odds ratios of the combined communication scores with the socio-demographic variables of nurses. Findings from this study showed that residents were rated poor on their communication skills when observed by younger nurses i.e., < 30 years (OR 1.95). Similarly, there was a significant difference in the communication skills of the residents with regards to frequency of interaction with the nurses (*p*-value of 0.001) and primary institution of undergraduate studies (*p*-value of < 0.001). Residents working in out-patient settings had better communication skills than those working in in-patient settings (*p*-value 0.106). Hence, family medicine residents were rated higher on their communication skills as compared to other specialties (*p*-value of 0.0001) (Refer to supplementary file 2).

**Table 2:** Crude and Adjusted Odds Ratio with 95% CI Using Ordinal Logistic Regression

	Crude OR (95% CI)	***p***-value	Adjusted OR (95% CI)	***p***-value
**Age**				
> 30	Ref	Ref	Ref	Ref
< 30 years	1.95 (1.08, 3.53)	0.027**	2.39 (1.27, 4.51)	0.007**
**Gender**				
Male	Ref	Ref		
Female	0.65 (0.28, 1.50)*	0.320		
**Interaction frequency**				
< 2	Ref	Ref	Ref	Ref
2–6	0.22 (0.09, 0.55)	0.001**	1.90 (0.90, 4.03)	0.090
> 6	0.51 (0.24, 1.08)	0.079	4.96 (1.96, 12.57)	0.001**
**Institution of Undergraduate Education**				
Public	Ref	Ref	Ref	Ref
Private	4.09 (1.97, 8.47)	< 0.0001**	3.62 (1.73, 7.55)	0.001**
**Years of Experience**				
< 5 years	Ref	Ref		
5–10 years	1.00 (0.51, 1.93)	0.993		
> 10 years	0.76 (0.35, 1.64)*	0.495		
**Setting of interaction**				
Outpatient	Ref	Ref	Ref	Ref
Inpatient	1.76 (0.88, 3.49)	0.106	1.19 (0.54, 2.62)	0.658
**Setting by Ward and Private**				
Ward	Ref	Ref		
Private	1.11 (0.29, 4.19)	0.867		

*for study characteristics that had a *p*-value of > 0.25 in the crude OR were not entered in the adjusted model

**p-value of < 0.05 considered as significant

These four socio-demographic variables were then entered in the stepwise adjusted logistic regression model. A significant difference in communication skills was seen in residents who were observed by nurses of a different age group, primary institution of undergraduate studies, and having increased interaction with residents. These are reported in Table 2.

Individual mean scores of nurses of residents’ communication skills based on the socio-demographic variables have been reported in Supplementary file 2. Younger nurses (aged < 30 years) were more likely to rate residents’ communication skills inadequate as compared to older ones (aged > 30 years). Similarly, residents, who were observed by female nurses, and interacted with nurses having > 5 years of experience were rated inadequate in their communication skills. Additionally, frequency, and duration of interaction with nurses was found to be inversely related to their rating of residents’ communication skills, where more interaction and hence, opportunity for observation led to poor rating of the residents. Furthermore, family medicine residents had the highest communication skills rating as compared to residents from other specialties.

### Qualitative results

In-depth interviews were conducted with nine nurses. Table 3 illustrates the demographic characteristics of the nurses who participated in the IDIs.

**Table 3:** Demographic characteristics of the IDI participants (*n* = 9)

Characteristic	n (%)
**Age (years)**	
Mean	32.5
Range	26–40
**Gender**	
Male	3 (33.33)
Female	6 (66.66)
**Years of Experience**	
Mean	9.3
Range	2–18
**Department**	
Surgery	2 (22.22)
Medicine	1 (11.11)
Pediatrics	2 (22.22)
Family medicine	2 (22.22)
Obstetrics/gynecology	2 (22.22)

Thematic analysis revealed two major themes and five subsequent subthemes. These are described as follows.

### Theme 1: Existing status of communication skills

#### 1.1 Deficient verbal and non-verbal communication

According to participants, residents tended to overuse medical jargon with patients which often left patients confused about their diagnosis, treatment and/or prognosis. This, in turn, led to patients repeatedly asking questions which would cause residents to behave harshly. Nurses also mentioned how most residents were unable to translate medical terminologies into the local language (Urdu) leading to a language barrier between the doctor and patient.

*“New residents sometimes use medical jargon or English terminologies, so the attendant does not understand it.”. (Surgical ward, 29y male).*

Some participants also mentioned that junior residents, who lacked adequate subject knowledge had trouble communicating effectively with patients.

With regards to non-verbal communication skills, participants particularly pointed to residents not maintaining adequate eye contact with patients which hindered successful rapport building.

*“Sometimes the residents are too busy writing in their files and do not maintain eye contact with the patient”. (Medicine, 35y female).*

Some participants also felt that the residents did not give adequate time to patients because of which patients and their attendants could not resolve their queries.

#### 1.2 Bias in inpatient counseling

It was reported that residents had a different attitude towards patients from different educational backgrounds. According to the participants, residents were more conscious and effortful in their counseling of educated patients as opposed to those who were not educated.

*“If the attendants and family members are educated, the residents and the consultants talk very carefully with them because they know that they are knowledgeable … those who are uneducated and don’t have a good idea, they are told things superficially”. (Gynecology, 40 y female).*

Nurses believed that residents should counsel all patients and attendants equally, regardless of their education level, since inadequate counselling resulted in some attendants calling the residents repeatedly.

#### 1.3 Challenging patient interactions

Nurses also shared their observations of the kind of patient-resident interactions that residents found challenging. These included communicating with patients who were knowledgable regarding diseases and would cross-question residents, and responding to angry patients. Some participants also noted that female residents felt hesitant in talking to male patients and vice versa.

*“Some patients are highly educated and have good know-how about diseases and treatment process, especially those having attendants who are hospital employees, doctors, nurses. These kinds of patients make the residents nervous and hesitant which leads them to not communicate effectively”. (Gynecology, 42y female).*

### Theme 2: Recommendations for improving patient-resident communication 

#### 2.1 simulation-based training

Participants suggested the introduction formal communication skills training for residents to improve their communication with patients. They highlighted the modalities for inculcating these skills which included the use of simulation-based training through which residents could improve their language, tone of voice, and non-verbal gestures while interacting with patients.

*“We should include simulation-based education where residents are shown hypothetical clinical scenarios and made to practice how to communicate with attendants of different personalities. This will make their communication clear and help them to explain medical jargon to ensure that the level of understanding remains on the same page”. (Family medicine, 27y female).*

#### 2.2 Troubleshooting difficult situations

Alongside teaching modalities, participants also identified specific aspects of communication skills to be taught to residents. They felt that residents needed to be equipped with local terminologies and translations of medical jargons to adequately counsel patients. They also highlighted that specialized communication skills were required with attendants of patients whose condition was not stable and that residents needed to be cognizant of such situations.

*“If the patient’s condition is deteriorating, then attendants find it difficult to trust the doctor. So, in that situation, communication should be better, clear, and realistic, and residents shouldn’t give false hope*.*”. (Surgical ward, 29y male).*

### Mixed methods findings

Integrating data from the quantitative and qualitative sources led to confirmation of the findings as illustrated in table 4.

**Table 4:** Joint display of quantitative and qualitative results

Quantitative data	Qualitative quotes	Mixed methods inferences
Barriers to effective resident-patient communication:		
Long working hours (78.24% agreement)	“During long duty hours in which residents are covering the night shift, their communication is not as effective.”	Confirmed Long working hours was the most highlighted barrier in the survey and also mentioned in the IDIs.
Human failings like stress and fatigue (67.19% agreement)	“If the patient flow is too much, sometimes residents behave harshly with the patients.”	Confirmed
Difficulty in understanding the patients’ language (56.99% agreement)	“In instances where there is a language barrier between the resident and patient, it becomes challenging for the residents to communicate.”	Confirmed According to participants, residents had difficulty interacting with patients who spoke a different cultural language. In such cases, the residents relied on translators for interacting with the patient.
Lack of subject knowledge required for fully explaining the modalities (56.48% agreement)	“If residents are not confident or competent or uncertain in knowledge then that becomes a barrier for them to communicate effectively with the attendants also.”	Confirmed Lack of adequate subject knowledge was cited as a barrier to residents effectively communicating with the patients. However, nurses in the IDIs indicated that as residents’ expertise and knowledge increased overtime, their communication also improved subsequently.

## Discussion

Nurses in our study were able to identify a significant lack of both verbal and non-verbal communication skills among residents which are essential for successful patient-resident interactions. This gap was significantly recognized by female nurses aged ≤30 years and nurses who received their undergraduate training at private institutions. In our cohort, family medicine residents were rated the highest in having adequate communication skills while interacting with patients, specifically in non-verbal communication, breaking bad news, and team dynamics. Interestingly, the findings also showed that the higher the interaction nurses had with residents, the lower were their ratings of residents’ communication skills. The major barriers identified by the participants for residents' inadequate communication skills included long working hours, immense stress, fatigue and lack of time to interact with patients. The qualitative findings were congruent with the quantitative results and further highlighted the areas of communication where residents need training. These included an overuse of medical jargon when communicating with patients, having a bias based on the patient’s literacy level, gender and inquisitiveness.

Our results showed that nurses believe patient centeredness to be the focal point in effective patient-resident communication encounters. Nurses and residents are profoundly involved in providing first-line care to patients [20], however, literature suggests an overall declining patient-centeredness and empathy in residents’ communication [21, 22]. Being in close coordination with each other, nurses have a leverage over other healthcare providers in observing patient-resident communication at multiple intervals. In our study, nurses perceived communication skills of family medicine residents higher than those in other specialties. This is consistent with results of Alsaad et.al. where patients were majorly satisfied by family medicine residents and rated them excellent in most items of the Communication Assessment Tool (CAT) [23]. However, studies conducted in Saudi Arabia reported surgery and internal medicine residents to outperform postgraduate trainees of other specialties in communication skills [24, 25].

Our results showed that majority of the time, residents did not consider the level of knowledge and prior information of patients regarding their condition before breaking bad news. A study conducted in Pakistan revealed a significant lack of knowledge regarding breaking bad news protocols amongst residents, [26] while others have highlighted a severe lack of assessment of the patient’s understanding and expectations by residents before delivering bad news [27, 28]. This evidence supports a dire need of residents’ training in communication skills.

In addition to emphasizing poor communication skills of residents, nurses in our study also identified the barriers which prevent residents in effectively communicating with their patients, specifically their long working hours leading to increased stress and fatigue. A systematic review found “high workload and fatigue” as one of the major barriers to communication in clinical settings [29]. Multiple other studies also specify residents’ stress and tiredness as contributing factors to ineffective communication with patients [30]. Similar to our results, Albahri et.al. reported limited time for interaction as one of the highest ranking barriers by both patients and physicians which hinders effective patient-doctor communication [31].

Our qualitative findings further aided in recognizing the aspects of residents’ communication which need improvement. One of the major issues highlighted was the overuse of medical jargon impeding patients’ comprehension. Recent research advancements have shown that communication skills must have some basic principles of information transfer for better patient outcomes and adherence to treatment [32–34]. These include the use of uncomplicated terminologies with minimal jargon, summarization of points with adequate repetitions, and an assessment of patient’s understanding [32–34]. Nurses also reported residents to prioritize taking notes regarding the patient’s history rather than maintaining eye contact with them. Similar observations extended to discussions that were of great emotional and social value to the patient. This practice of non-verbal communication results in delaying the disclosure of information by patients, adversely impacting patient outcomes [35, 36]. It is interesting to note that the literacy level of patients had a strong influence on residents’ communication with them, where educated patients were given more in-depth information. This behavior is broadly classified as “implicit bias”, [37] and results in disparities within the services provided to patients, with vulnerable populations often at a greater disadvantage than others [38].

Our findings identify the need for developing a communication skills curriculum within the postgraduate medical education program. Recognition of this need has led many institutions to adopt a curriculum for improving residents’ communication skills, the impact of which has been substantial [39–41]. This study highlighted several aspects of communication skills to be taught to residents, including breaking bad news, simplifying medical terminologies and eradicating the effect of implicit bias while interacting with patients. Similar to prior studies, [42, 43] simulation was identified as a prominent strategy in teaching these skills. This will help in bolstering the overall communication skills of residents, ultimately improving patient-resident encounters and satisfaction.

This study is the first of its kind undertaking a mixed-methods needs assessment of residents’ communication skills from the perspectives of nurses at the largest AMC in the country. The findings provide valuable insight into the need for developing and implementing a communication skills curriculum in the residency program. However, there are some limitations as well. This was a single center cross-sectional study which may limit generalizability of the findings. But given that many residents and nurses in this center hail from different settings from all over the country, our participants serve as a nationally representative sample and hence partial insight can be drawn from this study. Also, the initial self-assessment tool was modified so that nurses, as observers, can rate residents' communication skills that decreases bias. There were a limited number of nurses in the IDIs, however the qualitative findings were substantiated by the survey responses that gave some valuable insight regarding residents' communication skills from the lens of nurses.

Our findings indicate that resident-patient encounters are drastically affected by the residents' skills of communication and some institutional barriers such as long working hours and lack of time suggesting a need for improvement, To properly extricate aspects of residents’ communication skills that need the most improvement, a thorough evaluation of their overall interactions should be made. These can then be used to design a curriculum during residency which caters to the identified limitations and teach budding residents how to overcome them in clinical settings.

## Supplementary Information

**Additional file 1.**

**Additional file 2.**

**Additional file 3.**

## Data Availability

The datasets generated and/or analyzed during the current study have been included in the results section. A raw data file has been added in the supplementary material.
