# Supplementary material for: Leveraging the vantage point – exploring nurses’ perception of residents’ communication skills: a mixed-methods study
Source: BMC Med Educ. 2023 Mar 3;23:148. doi: 10.1186/s12909-023-04114-6 (PMC9985286; doi:10.1186/s12909-023-04114-6)
Supplement: Supplementary file 1 — Additional file 1. [file 12909_2023_4114_MOESM1_ESM.pdf]

CSI-P Questionnaire for Nurses to Assess Communication Skills of Medical Residents

Demographics:

Gender: Male Female Prefer not to Answer
Age:
Marital Status: Single Married Divorced Widowed
Current Assigned Specialty:
Current Assigned Subspecialty (if applicable):
Which unit are you currently assigned in? ______________ Ward/Clinic
How many years have you been a practicing nurse? ___________ Years
How many residents, on average, do you interact with residents on a daily basis?
0 <2 2-4 4-6 6-8 >8
Year of Completion of Undergraduate Education:
Name of Institution where Undergraduate Education was received:

Section A

A1. How often do the residents in your unit experience minor conflicts with a patient and/or attendants (caregivers/family members, people who accompany the patient) while fulfilling clinical responsibilities at your workplace?
[Minor conflict: an incident of unwarranted argument/debate resulting in *argumentative discussions, shouting, etc.* but not verbal abuse or physical violence]
A. Nearly daily
B. About once a week
C. About once a month
D. About once every 6 months
E. About once a year or less

A2. How often do residents in your unit experience major verbal conflicts (exchange of verbal abuse) with patients and/or attendants while fulfilling clinical responsibilities at your workplace?
A. Nearly daily
B. About once a week
C. About once a month
D. About once every 6 months
E. About once a year or less

A3. How many times have the residents in your unit encountered physical violence with patients and/or attendants while fulfilling clinical responsibilities at your workplace?

A. Four times or more
B. Three times
C. Two times
D. Once
E. None

A4. In your opinion, what proportion of doctor-patient conflicts can be avoided by good communication practice of the residents (i.e., patiently listening to your patients, fully explaining the nature and yield of investigations, benefits of treatment, course and prognosis of the disease).

A. Almost all
B. About 75%
C. About 50%
D. About 25%
E. None

Section B: Assessment of Resident's Communication Skills

Please rate the extent to which you think the residents in your unit practice the following:

A. Always
B. Often
C. Sometimes
D. Occasionally
E. Rarely

Effective communication has three basic components: verbal, non-verbal and paraverbal. The verbal component deals with the content of the message including the selection of words. The non-verbal component includes body language like posture, gesture, facial expression, and spatial distance. The paraverbal component includes tone, pitch, pacing, and volume of the voice. Questions 1–5 mostly address points pertaining to these aspects:

The medical residents in my unit:

B1. Greet a patient warmly upon meeting them with a smile/say hello.
B2. Prefer to address the patient by name during history taking/examination or interview.
B3. Make eye contact during conversation or interview.
B4. Try to avoid any interruption such as taking calls or checking messages while a patient is talking.
B5. Pay attention to non-verbal cues like gestures and facial expressions of the patients.

Content and setting of discussion/interview sessions is an important aspect of communication with patients/attendants in the outpatient department/indoor setting. Questions 6–12 mostly address points pertaining to this aspect:

The medical residents in my unit:

B6. Ensure privacy while conducting interview/discussion sessions with patients/attendants.

B7. Prefer simple language and avoid medical jargon and abbreviations.

B8. Explain the nature, course, and prognosis (both short term and long term) of the disease in detail.

B9. Explain in detail regarding the necessity and feasibility of expensive investigations and their effect on the course and outcome of the disease.

B10. Explain in detail regarding various treatment options available and their effect on the course and outcome of the disease.

B11. Involve the patient in the decision-making regarding the choice of investigation and treatment.

B12. Ask the patient if he/she would like additional information before concluding the interview.

Communicating with patients and attendants in indoor/high dependency unit/intensive care unit is challenging. Questions 13–18 are mostly related to the aspect of communicating with patients/attendants in this setting:

The medical residents in my unit:

B13. Try to answer the queries of the attendants which they gather from the internet or other sources by giving better references.

B14. Emphasize the dynamic nature of the disease while discussing the daily progress of the patients.

B15. Inform the patient about the course and prognosis of the disease multiple times during a day with the attendants when a patient is seriously ill.

B16. Talk to attendants and discuss in detail after visiting the patient's bed.

B17. Take consent from patients/attendants themselves.

B18. Take consent from patients/attendants after detailed discussion.

Bad news means any information that has the potential to have a devastating influence on one's life. Breaking bad news is challenging for any doctor. Questions 19 to 23 deal with the different steps involved in the practice of breaking bad news.

The medical residents in my unit:

B19. Plan in advance and mentally rehearse the act of disclosure before breaking bad news.

B20. Tend to assess relative's/patient's knowledge and attitude by asking open-ended questions before breaking bad news.

B21. Tend to give information in small portions rather than doing so abruptly while breaking bad news.

B22. Address/attend to patient's emotional reaction with full patience after breaking bad news.

B23. Discuss the future plan of treatment with the patients and/or attendants after breaking bad news.

Team dynamics is important in the success of a treating team. The practice of good communication skills with colleagues, nurses, paramedical staffs and other supporting staff is extremely important. Questions 24 to 29 address this aspect:

The medical residents in my unit:

B24. Display appropriate courtesy while communicating with nurses, paramedical staff, and other supporting staff.

B25. Highlight that the role and responsibility of nurses, paramedical staff, and other supporting staff is equally important while communicating with them.

B26. Avoid criticising colleagues or having debates in front of patients or attendants.

B27. Teach nurses, paramedical staff, and other supporting staff the principles of management of commonly encountered diseases in the ward to motivate them.

B28. Regularly express appreciation for nurses, paramedical staff, and other supporting staff as a part of giving feedback.

B29. Don’t hesitate to give positive criticism/constructive suggestion as a part of giving feedback to their subordinates and supporting healthcare staff.

Section C: Barriers to Practicing Good Communication Skills

In your opinion, to what extent do you agree/disagree that the following barriers prevent the residents from practicing good communication skills (i.e., listening to patients adequately, explaining in detail the nature and yield of investigations, course and prognosis of the disease, benefits of treatment, etc.):

- **A. Strongly Agree**
- **B. Agree**
- **C. Neutral**
- **D. Disagree**
- **E. Strongly Diagree**

C1. Lack of insight

C2. Lack of time

C3. Difficulty in understanding the patient’s language

C4. Human failings like stress and fatigue

C5. Infrastructural deficits like lack of proper place for discussion, overcrowding.

C6. Long working hours

C7. Lack of subject knowledge required for fully explaining the modalities of diagnosis, treatment options, or prognosis

C8. Lack of training in communication skills

CSI-P Qualitative Semi-Structured Interview Guide

Name: Gender:
Designation/Dept: Age:

Section A (General):

1. What do you think characterizes effective communication between a physician (resident in this case) and their patient?
2. Can you comment on the relevance of communication skills in medical training?
3. Have you ever felt that a lack of communication skills was a barrier in patient care? Would you like to provide an anecdotal example of the above?
4. Do you think a formal curriculum would help individuals improve their communication skills?

Section B (Nurses):

1. On average, how would you appraise the resident's communication skills?
2. Can you identify specific shortcomings in the communication skills of the residents?
3. Do you think the residents lack empathy or are unable to show empathy to their patients and their families while interacting with them?
4. Does the tone of voice affect physician – patient communication?
5. Do you think the residents have an appropriate tone while interacting with patients?
6. Do you think the residents' body language plays a role in how patients perceive them?

7. Do you think that their body language and eye contact is appropriate for interacting with patients?

8. What is the most difficult interaction you have witnessed of a resident with a patient?
