# Supplementary material for: Leveraging the vantage point – exploring nurses’ perception of residents’ communication skills: a mixed-methods study
Source: BMC Med Educ. 2023 Mar 3;23:148. doi: 10.1186/s12909-023-04114-6 (PMC9985286; doi:10.1186/s12909-023-04114-6)
Supplement: Supplementary file 2 — Additional file 2. [file 12909_2023_4114_MOESM2_ESM.pdf]

Supplementary File 2

**Table S1: Nurse's interaction with the residents**				
	A1. How do the residents in your unit experience minor conflicts with a patient	A2. How do residents in your unit experience major verbal conflicts (exchange	A3. How many times have the residents in your unit encountered physical violence	A4. In your opinion, what proportion of doctor-patient conflicts can be avoided
**Characteristics**				
**Age**				
≤30 years	3.10 (1.46)	3.87 (1.31)	4.38 (1.11)	1.86 (1.04)
> 30 years	3.57 (1.63)	4.15 (1.35)	4.70 (0.60)	2.05 (1.36)
p-value	0.0191*	0.0732	0.0076*	0.1354
**Institutions**				
Private	3.22 (1.54)	3.91 (1.35)	4.57 (0.90)	1.84 (1.11)
Public	3.68 (1.61)	4.29 (1.26)	4.43 (0.97)	2.31 (1.45)
p-value	0.0914	0.1022	0.3803	0.0228*
**Years of Experience**				
<5 years	3.14 (1.53)	3.74 (1.45)	4.38 (1.12)	1.88 (1.14)
5-10 years	3.29 (1.54)	3.92 (1.36)	4.61 (0.86)	1.97 (1.19)
>10 years	3.66 (1.61)	4.52 (0.96)	4.64 (0.60)	2.02 (1.34)
p-value	0.1966	0.0059*	0.214	0.8258
*p-value of <0.05				

**Table S2: Non-verbal Components of Communication**					
	B1. Greet a patient warmly upon meeting them with a	B2. Prefer to address the patient by name during history taking/examination	B3. Make eye contact during conversation or interview.	B4. Try to avoid any interruptions such as taking calls or checking messages while	B5. Pay attention to non-verbal cues like gestures and facial

	smile/say hello.			communicating with the patients	expressions of the
**Characteristics**					
**Institutions**					
Private	4.24 (1.03)	4.40 (0.85)	4.27 (0.95)	3.70 (1.22)	4.83 (1.15)
Public	4.45 (0.90)	4.68 (0.51)	4.48 (0.63)	4.38 (0.94)	4.18 (1.16)
p-value	0.2176	0.0462*	0.0084*	0.0008*	0.0796
**Assigned Specialty**					
Family Medicine (n= 16)	4.5 (0.63)	4.37 (0.61)	4.5 (0.73)	4.27 (0.80)	4.18 (0.83)
Internal Medicine (n= 22)	4.27 (1.07)	4.5 (0.74)	4.5 (0.67)	4.13 (0.71)	3.95 (1.25)
Obs/Gyn (n= 93)	4.44 (0.89)	4.68 (0.62)	4.50 (0.77)	3.95 (1.24)	3.91 (1.18)
Pediatric (n=27)	4.22 (1.08)	4.44 (0.69)	4.22 (1.28)	3.96 (1.15)	4.18 (1.11)
Surgery (n=27)	3.88 (1.15)	3.88 (1.18)	4.00 (0.96)	3.03 (1.25)	3.44 (1.28)
Others (n= 8)	3.75 (1.48)	4.12 (0.83)	3.87 (1.12)	3.37 (1.18)	3.87 (0.64)
p-value	0.0871	0.0002*	0.0595	0.0015*	0.2367
**Interaction Frequency**					
<2 interactions	4.53 (0.66)	4.60 (0.54)	4.69 (0.55)	4.16 (1.06)	4.34 (0.75)
2-4 interactions	4.24 (0.98)	4.51 (0.65)	4.28 (0.81)	3.81 (1.08)	3.84 (1.16)
4-6 interactions	4.54 (0.86)	4.59 (0.76)	4.48 (0.93)	3.83 (1.36)	4.16 (0.98)
6-8 interactions	3.76 (1.52)	4.11 (1.16)	3.82 (1.28)	3.47 (1.32)	3.82 (1.07)
>8 interactions	4.00 (1.13)	4.19 (1.13)	4.23 (1.06)	3.76 (1.33)	3.07 (1.54)
p-value	0.0172*	0.0641	0.0073*	0.3087	0.0002*

Table S3: Content and setting of discussion/interview sessions

	B6. Ensure privacy while conducting interview/discussion sessions with patients/	B7. Prefer simple language and avoid medical jargon and abbreviations	B8. Explain the nature, course, and prognosis (both short term and long term)	B9. Explain in detail the necessity and feasibility of expensive	B10. Explain in detail regarding various treatment options available and their	B11. Involve the patient in the decision-making regarding the choice of investigation	B12. Ask the patient if he/she would like additional information before concluding sessions
**Characteristics**							
**Age**							
<=30 years	4.27 (1.04)	4.07 (1.11)	4.03 (1.11)	3.92 (1.23)	4.05 (0.94)	4.08 (1.08)	3.98 (1.21)
> 30 years	4.43 (0.89)	4.33 (0.86)	4.33 (0.82)	4.11 (1.15)	4.15 (0.96)	4.27 (0.98)	4.31 (0.82)
p-value	0.1326	0.038*	0.019*	0.1251	0.2501	0.1122	0.0139*
**Institutions**							
Private	4.26 (1.01)	4.13 (1.01)	4.10 (1.00)	3.93 (1.17)	4.01 (0.97)	4.10 (1.05)	4.02 (1.09)
Public	4.63 (0.80)	4.43 (0.94)	4.43 (0.94)	4.29 (1.24)	4.41 (0.81)	4.40 (0.97)	4.54 (0.81)
p-value	0.0281*	0.0854	0.0576	0.0774	0.015*	0.0912	0.0036*
**Assigned Specialty**							
Family Medicine (n= 16)	4.18 (0.91)	4.37 (0.71)	4.37 (0.71)	4.06 (0.85)	4.00 (0.81)	4.18 (0.98)	4.37 (0.61)
Internal Medicine (n= 22)	4.04 (1.17)	4.22 (1.15)	3.86 (1.12)	3.81 (1.25)	4.04 (1.13)	4.00 (1.23)	3.81 (1.46)
Obs/Gyn (n= 93)	4.59 (0.79)	4.36 (0.88)	4.37 (0.84)	4.24 (1.05)	4.29 (0.81)	4.30 (0.96)	4.31 (0.89)

Pediatric (n=27)	4.55 (0.75)	4.44 (0.93)	4.51 (0.89)	4.40 (1.18)	4.40 (0.79)	4.48 (0.89)	4.51 (0.75)
Surgery (n=27)	3.85 (1.29)	3.40 (1.21)	3.51 (1.22)	3.25 (1.31)	3.44 (1.08)	3.66 (1.20)	3.44 (1.31)
Others (n= 8)	3.75 (1.03)	3.75 (0.70)	3.5 (0.92)	3.00 (1.30)	3.5 (1.06)	3.87 (0.83)	3.62 (0.91)
p-value	0.0012*	0.0002*	0.0001*	0.0002*	0.0002*	0.0411*	0.0003*
**Interaction Frequency**							
<2 interactions	4.74 (0.53)	4.46 (0.76)	4.44 (0.79)	4.41 (0.95)	4.27 (0.90)	4.48 (0.76)	4.41 (0.76)
2-4 interactions	4.22 (0.99)	4.11 (0.97)	4.18 (0.90)	3.87 (1.14)	4.08 (0.91)	4.05 (1.01)	4.01 (1.13)
4-6 interactions	4.54 (0.80)	4.37 (0.95)	4.32 (0.97)	4.32 (1.02)	4.13 (0.97)	4.10 (1.32)	4.24 (1.09)
6-8 interactions	3.94 (1.14)	4.39 (1.19)	3.52 (1.41)	3.41 (1.73)	3.88 (1.16)	4.29 (0.98)	4.11 (1.16)
>8 interactions	4.03 (1.34)	3.92 (1.29)	3.96 (1.07)	3.69 (1.25)	3.96 (0.95)	4.00 (1.01)	3.88 (1.10)
p-value	0.0039*	0.1009	0.0143*	0.0049*	0.5576	0.2064	0.2119
*p-value of <0.05							

**Table S4: Communicating with Patients/ Attendants in Clinical Settings**						
	B13. Try to answer the queries of the attendants which they gather from the interview	B14. Emphasize the dynamic nature of the disease while discussing the daily prognosis	B15. Inform the patient about the course and prognosis of the disease multiple	B16. Talk to attendants and discuss in detail after visiting the patient's bed.	B17. Take consent from patients/attendants themselves.	B18. Take consent from patients/attendants after a detailed discussion
**Characteristics**						
**Institutions**						

Private	3.93 (1.07)	4.06 (0.87)	3.97 (1.11)	3.95 (1.03)	4.25 (1.06)	4.20 (1.03)
Public	4.5 (0.84)	4.54 (0.69)	4.5 (0.73)	4.40 (0.84)	4.56 (0.69)	4.36 (0.94)
p-value	0.0018*	0.0009*	0.0039*	0.0094*	0.0667	0.3727
**Assigned Specialty**						
Family Medicine (n= 16)	4.18 (0.91)	4.31 (0.70)	3.87 (1.36)	4.06 (1.12)	4.00 (1.36)	4.06 (1.06)
Internal Medicine (n= 22)	3.95 (1.04)	4.13 (0.94)	3.86 (1.45)	4.27 (1.07)	4.59 (0.5)	4.27 (1.03)
Obs/Gyn (n= 93)	4.10 (1.05)	4.26 (0.78)	4.24 (0.88)	4.13 (0.92)	4.37 (0.89)	4.32 (0.89)
Pediatric (n=27)	4.59 (0.69)	4.48 (0.64)	4.44 (0.75)	4.37 (0.74)	4.14 (1.16)	4.51 (0.80)
Surgery (n=27)	3.70 (1.10)	3.62 (0.96)	3.62 (1.11)	3.48 (1.18)	4.40 (0.97)	3.92 (1.35)
Others (n= 8)	3.12 (1.35)	3.62 (1.18)	3.87 (1.24)	3.5 (1.06)	4.00 (1.60)	3.75 (1.28)
p-value	0.004*	0.0013*	0.0304*	0.0079*	0.3708	0.1778
p-value	0.8365	0.9852	0.9921	0.8875	0.3667	0.3281
*p-value of <0.05						

Table S5: Breaking Bad News

	B19. Plan in advance and mentally rehearse the act of disclosure before breaking	B20. Tend to assess relative's/patient's knowledge and attitude by asking open-e	B21. Tend to give information in small portions rather than doing so abruptly	B22. Address/attend to patient's emotional reaction with full patience	B23. Discuss the future plan of treatment with the patients and/or attendants
**Characteristics**					
**Institutions**					
Private	3.70 (1.18)	3.83 (1.11)	3.90 (1.06)	3.90 (1.12)	4.04 (0.98)
Public	4.28 (0.94)	4.54 (0.62)	4.45 (0.72)	4.56 (0.58)	4.54 (0.72)
p-value	0.0006*	0.0001*	0.0017*	0.0002*	0.0021*
**Assigned Specialty**					

Family Medicine (n= 16)	3.21 (1.78)	3.68 (1.25)	3.68 (1.25)	3.75 (1.23)	3.87 (1.25)
Internal Medicine (n= 22)	3.54 (1.18)	3.72 (1.31)	3.90 (1.15)	4.00 (1.34)	3.86 (1.12)
Obs/Gyn (n= 93)	4.11 (0.95)	4.12 (0.93)	4.12 (0.93)	4.13 (0.92)	4.35 (0.73)
Pediatric (n=27)	4.22 (0.89)	4.48 (0.70)	4.48 (0.64)	4.48 (0.64)	4.44 (0.64)
Surgery (n=27)	3.55 (1.25)	3.74 (1.12)	3.81 (1.14)	3.74 (1.28)	3.77 (1.25)
Others (n= 8)	3.00 (1.30)	3.00 (1.30)	3.12 (1.12)	3.5 (1.19)	3.62 (1.06)
p-value	0.0005*	0.0021*	0.0069*	0.0514	0.0041*
*p-value of <0.05					

Table S6: Team Dynamics

	B24. Display appropriate courtesy while communicating with nurses, paramedical s	B25. Highlight that the role and responsibility of nurses, paramedical staff, an	B26. Avoid criticizing colleagues or having debates in front of patients or attendants	B27. Teach nurses, paramedical staff, and other supporting staff the principles	B28. Regularly express appreciation for nurses, paramedical staff,	B29. Don't hesitate to give positive criticism/constructive suggestions as a part
Characteristics						
Gender						
Female	4.18 (0.93)	4.04 (1.06)	3.86 (1.13)	3.86 (1.21)	3.65 (1.32)	3.95 (1.09)
male	4.03 (1.24)	3.84 (1.31)	3.16 (1.65)	3.5 (1.36)	3.53 (1.42)	3.42 (1.33)
p-value	0.489	0.4003	0.3192	0.1695	0.6811	0.0281*
Age						
<=30 years	3.97 (1.14)	3.80 (1.24)	3.61 (1.25)	3.58 (1.32)	3.42 (1.38)	3.66 (1.25)
> 30 years	4.36 (0.69)	4.25 (0.85)	4.06 (0.99)	4.07 (1.09)	3.88 (1.22)	4.11 (0.94)
p-value	0.0021*	0.0022*	0.0033*	0.0028*	0.0085*	0.0025*
Institutions						

Private	4.10 (0.97)	3.91 (1.10)	3.71 (1.11)	3.76 (1.20)	3.53 (1.30)	3.77 (1.14)
Public	4.36 (0.94)	4.36 (1.01)	4.15 (1.23)	4.00 (1.34)	4.02 (1.33)	4.22 (1.03)
p-value	0.1157	0.0162*	0.031*	0.2702	0.0302*	0.021*
Assigned Specialty						
Family Medicine (n= 16)	4.27 (0.80)	4.31 (0.74)	4.31 (0.79)	3.93 (1.28)	4.12 (0.95)	4.00 (0.96)
Internal Medicine (n= 22)	3.81 (1.33)	3.72 (1.31)	3.36 (1.39)	3.22 (1.50)	3.09 (1.50)	3.22 (1.47)
Obs/Gyn (n= 93)	4.27 (0.87)	4.25 (0.95)	3.97 (1.06)	4.16 (1.07)	3.94 (1.18)	4.21 (0.89)
Pediatric (n=27)	4.40 (0.74)	4.25 (0.98)	4.18 (1.11)	4.00 (1.14)	4.07 (1.10)	4.11 (1.08)
Surgery (n=27)	3.74 (1.05)	3.14 (1.16)	3.22 (1.15)	3.03 (1.15)	2.48 (1.34)	3.11 (1.21)
Others (n= 8)	3.87 (1.12)	3.5 (1.19)	3.25 (1.16)	3.25 (1.03)	3.12 (0.99)	3.37 (0.91)
p-value	0.0284*	0.00*	0.001*	0.0001*	0.000*	0.000*
Interaction Frequency						
<2 interactions	4.48 (0.88)	4.25 (0.97)	4.25 (0.97)	4.32 (0.94)	3.95 (1.21)	4.13 (1.08)
2-4 interactions	4.08 (0.89)	4.08 (0.91)	3.57 (1.22)	3.58 (1.24)	3.44 (1.35)	3.74 (1.15)
4-6 interactions	4.18 (1.04)	3.91 (1.27)	3.86 (1.05)	3.72 (1.38)	3.72 (1.34)	3.94 (1.20)
6-8 interactions	4.11 (1.11)	3.70 (1.40)	4.00 (1.22)	3.47 (1.41)	3.52 (1.46)	3.70 (1.35)
>8 interactions	3.80 (1.02)	3.76 (1.21)	3.65 (1.19)	3.96 (1.11)	3.61 (1.29)	3.84 (0.92)
p-value	0.0654	0.2584	0.0338*	0.0195*	0.3773	0.4363
*p-value of <0.05						
